# National study for multidisciplinary outpatient oncological rehabilitation: online survey to support revised quality and performance criteria

**DOI:** 10.1007/s00520-020-05913-z

**Published:** 2020-12-08

**Authors:** Graham Dorey, Sophie Cabaset, Aline Richard, Anna Dehler, Daisy Kudre, Beate Schneider-Mörsch, Nicolas Sperisen, Margareta Schmid, Sabine Rohrmann

**Affiliations:** 1 Division of Chronic Disease Epidemiology, Epidemiology, Biostatistics and Prevention Institute, University of Zurich, Hirschengraben 82, CH-8001 Zurich, Switzerland; 2 Krebsliga Schweiz, Effingerstrasse 40, CH-3001 Bern, Switzerland

**Keywords:** Cancer rehabilitation, Switzerland, Outpatient, Multidisciplinary, Survey

## Abstract

**Purpose:**

More and more people survive cancer, but the disease and its treatment often lead to impairment. Multidisciplinary ambulatory oncological rehabilitation (OR) programs have thus been developed. SW!SS REHA, the organization of major Swiss rehabilitation clinics, has defined ambulatory OR quality criteria for its members (about 50% of the Swiss rehabilitation capacity). However, SW!SS REHA criteria are not fully implemented and/or interpreted differently by different specialties or in different linguistic regions in Switzerland. The aim of our study was to carry out an online survey of existing outpatient programs to define quality criteria for an ideal OR program in Switzerland.

**Methods:**

A mixed methods approach was used for the survey—qualitative and quantitative. The qualitative part consisted of a guided discussion with OR experts and the quantitative part of an online survey. The quantitative part comprised the development and evaluation of an online questionnaire. It served to record the opinions of OR centers in Switzerland on the desired situation of outpatient rehabilitation.

**Results:**

Eighteen OR centers and 71 (49.7% response rate) OR actors participated in the online survey. The survey results indicate that some of the SW!SS REHA quality and performance criteria only partially match with the desired OR criteria for Switzerland. Key disparities occur particularly in the program design and structure and specifically around how many interventions are required to constitute an OR program, the extent of standardization versus individualization of the program, i.e., how many and which modules in a program should be obligatory, and finally the duration and intensity of the program. The online survey did not generate any statistical evidence that OR requirements vary significantly between different linguistic regions and among different specialties.

**Conclusions:**

Cancer patients are heterogeneous with respect to cancer type, prognosis, and disability level, such that a standard program cannot be uniformly applied. Therefore, a flexible program is required with few mandatory modules and additional individual modules to achieve the threshold number of modules that would constitute a multidisciplinary OR program. Intensity and frequency of OR needs to consider the health state of the participants. The results indicate a need to modify some of the existing SW!SS REHA criteria to ensure that more patients can gain access and benefit form evidence-based OR interventions. Furthermore, the survey provides important findings so that the existing OR offer can be improved with the goal that OR centers will be able to be quality certified in the future.

**Supplementary Information:**

The online version contains supplementary material available at 10.1007/s00520-020-05913-z.

## Introduction

More and more people in Switzerland are diagnosed with cancer due to increasing life expectancy, but thanks to improved early detection and better treatment options, more and more people are surviving a cancer diagnosis. For 2015, the number of cancer survivors in Switzerland was estimated at 317,000 and are continuously increasing [1]. However, the disease and its treatment often lead to physical, psychological and social impairments. Multidisciplinary oncological rehabilitation programs have been shown to improve the quality of life and participation of people with cancer and facilitated their reintegration into daily and working life treatment [2]. In addition to a specialist physician in rehabilitation medicine, these kinds of program include paramedic disciplines, e.g. physiotherapists, occupational therapists, psychologists, social counselors, nurses and speech therapists [3]. As a result of the success of these programs, cancer-specific rehabilitation programs have been developed in various countries such as the Netherlands [4], Australia [5], Canada [6], and Switzerland [7]. However, only a minority of countries with cancer rehabilitation programs have national guidelines for cancer rehabilitation, which are supposed to set standards for the programs [8]. It remains, therefore, unclear whether or which multidisciplinary cancer rehabilitation programs are an effective intervention for cancer survivors [9].

In Switzerland, cancer-specific rehabilitation programs have mainly taken place in an in-hospital setting. Because of the increasing number of cancer patients, changing patient wishes and changes in financial conditions, the number of centers offering oncological rehabilitation in an outpatient setting in Switzerland has steadily increased over recent years. However, these programs are inconsistent in their design and often poorly coordinated. SW!SS REHA is an organization of major Swiss rehabilitation clinics, covering about 50% of the Swiss rehabilitation capacity of any medical specialty (http://www.swiss-reha.com/). It has previously defined ambulatory oncological rehabilitation (OR) quality criteria for its members. However, it has been observed that the SW!SS REHA criteria are not fully implemented and/or interpreted differently by different specialties or in different linguistic regions in Switzerland (Dehler A. et al., submitted). The aim of this project was therefore to establish the basis for the desired outpatient OR program from the providers’ point of view and, if necessary, for recommendations for adjustments to the SW!SS REHA criteria.

## Materials and methods

An online questionnaire was developed based on a previous survey (Dehler A. et al., submitted) and a guided discussion among Swiss outpatient OR providers [8] aiming at collecting the opinions and attitudes of professionals working in outpatient OR programs in Switzerland with focus on the ideal program design of the future and reflection on the current SW!SS REHA criteria. The questionnaire was divided into the following main sections:

- Management and organization: who should lead and co-ordinate the program; information flow and patient management; required expertise of those involved
- Screening requirements for admission: choice of rehabilitation instruments to evaluate rehabilitation needs and measure functional deficit
- Program design: rehabilitation program design considerations for standardized and individual programs depending whether the program begins during or after completion of the acute oncological treatment; which modules should be on offer; how much rehabilitation is feasible for patients during or after their acute treatment
- Financing and certification: program financing and reimbursement models; advantages of a certification for the OR centers

Most of the questions were closed with both single choice and multiple-choice answers (appendix 1). As necessary, some open questions were also included in the questionnaire. All questions were mandatory apart from four free text questions.

In a first phase, the questionnaire was reviewed by members of the project team and tested. The revised version of the survey was tested once again. The German version was then translated into French and Italian and the translated questionnaires were cross-checked with respect to language.

All 18 ambulatory OR centers operating in March 2019 in Switzerland were invited to participate. All OR centers provided a list of employees, which were invited by e-mail to participate in the online survey (*n* = 145). The invitees of the online survey included oncologists, internists, physiotherapists, exercise/sports therapists, nutritionists, psycho-oncologists, social counselors, complementary medicine specialists, nurses, pain therapists, pastoral care, somatic therapists, and OR coordinators. The survey was conducted in April 2019 using “Survey Monkey ®.”

Based on the results of the first fact-based survey (Dehler A. et al., submitted), a guided discussion [8] and the different perspectives of the OR centers and actors, it was decided to analyze the results of the online survey with three language region variables (CH-D, CH-F, CH-T) and by three professional groups. These included the two largest participating groups consisting of physiotherapists and oncologists as well as a third mixed group of all the other participating specialists.

Data were analyzed using MS Excel. A descriptive statistical analysis of the survey results was conducted. As the questions in the questionnaire were frequently of a single choice nature and the variables to analyze were categorical, the chi-squared test was used to test for differences between subgroups of the study population. No test was conducted if multiple replies per question were possible.

## Results

### Characteristics of survey responders and non-responders

From the 145 individually identified OR professionals, 71 (49.0%) responded. Among the responders to the survey (Table 1), the largest professional group of responders were the physiotherapists (32.4%) followed by the oncologists (18.3%). The remaining specialties were combined for analysis purposes into an “other” group of responders (49.3%). In the three language regions (Table 2), there were differences in the response rates ranging from 55.8% in the D-CH to 36.1% in the F-CH. For the different specialties, the response rates ranged from 92.9% for the oncologists, 65.7% for the physiotherapists, to 36.4% for the “other” group.

**Table 1:** Online survey responder list/recipient list/response rate by specialty

Specialty	Responders	Recipients	Response rate (%)
Occupational therapy	2	5	40.0
Nutrition consultation	5	17	29.4
Complementary medicine	0	7	0.0
Management/coordination/administration	4	15	26.7
Oncology	13	14	92.9
Care/nursing	2	5	40.0
Physiotherapy	23	35	65.7
Psychotherapy/psychology/psychooncolgy	3	13	23.1
Pain therapy	0	2	0.0
Pastoral care	1	2	50.0
Social counselling	6	11	54.5
Sports, exercise therapy	7	5	140.0
Somatotherapy	0	1	0.0
Other	5	13	38.5
Total	71	145	49.0

**Table 2:** Response rates for the different specialties and language regions

	German-speaking	French-speaking	Italian-speaking	Total
Physiotherapist	91.7% (11/12)	30.0% (3/10)	69.2% (9/13)	65.7% (23/35)
1. Oncologist	70.0% (7/10)	133.3% (4/3)*	200.0% (2/1)*	92.9% (13/14)
2. Other	45.5% (25/55)	26.0% (6/23)	22.2% (4/18)	36.4% (35/96)
3. All specialties	55.8% (43/77)	36.1% (13/36)	46.8% (15/32)	49.0% (71/145)

*Participants were free to choose their specialty; some participants hold training in more than one specialty

### Management and organization

Of the participants, 91.4% stated a medical doctor should lead the OR (Table 3). Furthermore, for 57.7% of the respondents, it should be the oncologist. The free text answers in the questionnaire provided some insights on why the oncologist was viewed as the preferred director of the rehabilitation. Oncologists understand the different rehabilitation problems of oncological patients very well and then pass them on to the appropriate rehabilitation specialists. Central is the competence in supportive oncology, i.e., dealing with side effects of cancer therapies.

**Table 3:** Management and organization by medical specialty

	Oncologist	Physiotherapist	Other	Total
	*n* = 13	*n* = 23	*n* = 35	*n* = 71
Who should be the medical director of outpatient oncological rehabilitation? (multiple selection possible)				
Oncologist	72.2%	61.8%	50.0%	57.7%
AIM	11.1%	5.9%	13.5%	10.6%
PMR	16.7%	29.4%	21.2%	23.1%
Other specialist*	0.0%	2.9%	7.7%	4.8%
Do not know	0.0%	0.0%	7.7%	3.8%
How much rehabilitation experience is needed to take over the medical management, if not PMR (physical and medical rehabilitation physician)?				
None	23.1%	4.3%	14.3%	12.7%
Up to 1 year	15.4%	4.3%	5.7%	7.0%
Between 1 and 2 years	15.4%	34.8%	8.6%	18.3%
2 years or more	23.1%	26.1%	25.7%	25.4%
I do not know	23.1%	30.4%	45.7%	36.6%
*P* = 0.81				
How should the flow of information between specialists be supported? (multiple selection possible)				
KLS rehab logbook	13.0%	16.3%	23.2%	18.9%
Via e-mail	26.1%	20.9%	12.5%	18.0%
Formalized report	8.7%	9.3%	8.9%	9.0%
Electronic patient dossier	39.1%	39.5%	41.1%	40.2%
Other	13.0%	11.6%	12.5%	12.3%
I do not know	0.0%	2.3%	1.8%	1.6%
How often should an oncological rehabilitation team meeting take place?				
Once a week	15.4%	17.4%	11.4%	14.1%
Once a month	53.8%	43.5%	42.9%	45.1%
Once per program	7.7%	8.7%	22.9%	15.5%
Meeting not necessary	23.1%	26.1%	22.9%	23.9%
Other	0.0%	4.3%	0.0%	1.4%
*P* = 0.81				

*Family doctor, nurse, occupational therapist, physiotherapist, radiation oncologist, hematologist, psycho-oncologist (specialist for psychiatry and psychotherapy), rehabilitation physicians, any qualified specialist, psychosomatics. *AIM*, general physician; *PMR*, rehabilitation physician; *KLS*, Swiss Cancer League

There is no clear consensus regarding the length of experience necessary to lead the management of the OR. 60.9% of physiotherapists and 48.5% of oncologists considered at least 1 year of experience is necessary. Only one quarter of respondents considered that a full 2 years of experience are necessary. According to the free text answers, it was considered that after 1 year of experience, a certain understanding of OR has been achieved and adequate knowledge acquired to be able to lead the service.

The information flow for the OR team coordination should be supported ideally through an electronic patient dossier (43.7%). To the question, how often a rehabilitation team discussion should take place, the most frequently given response was once a month (45.1%).

### Screening instruments

Regarding generic screening instruments to measure the need for rehabilitation, there was no dominant instrument and the preferences were split between Functional Capacity Assessment (EFL) and/or Edmonton Symptom Assessment Score (ESAS) and/or WHO Disability Assessment Schedule (WHODAS) II and/or Eastern Cooperative Oncology Group (ECOG)/Karnofsky or adapted ECOG, and/or distress thermometer (Table 4). From the free text analysis, it was reported that none of these current instruments are suitable for diagnosing multimodal functional deficits that indicate an indication for inpatient or outpatient oncological rehabilitation.

**Table 4:** Screening Instruments. According to medical specialty

	Oncologist	Physiotherapist	Other	Total
	*n* = 13	*n* = 23	*n* = 35	*n* = 71
Which generic instruments do you consider useful for assessing rehabilitation needs? (multiple selection possible)				
ESAS score, if necessary WHODAS II	19.4%	21.1%	13.5%	17.4%
ECOG/Karnofsky or adapted ECOG	29.0%	13.2%	3.8%	13.2%
Evaluation of Functional Performance (EFL)	19.4%	23.7%	23.1%	22.3%
Distress thermometer	19.4%	13.2%	9.6%	13.2%
Further assessments	9.7%	7.9%	7.7%	8.3%
I do not know	3.2%	21.1%	42.3%	25.6%
Do you consider specialist instruments for measuring progress and goal achievement in your field to be useful? E.g. 6-minute walking test, timed get up and go, HADS, NRS, etc.				
Yes	76.9%	100.0%	62.9%	77.5%
No	15.4%	0.0%	11.4%	8.5%
I do not know.	7.7%	0.0%	25.7%	14.1%
*P* = 0.01				

The view on the value of specialty specific instruments for measuring progress and goal achievement across all specialist groups was three quarters in favor (77.5%). Among the three specialty groups, the value of specialty specific instruments was perceived differently (Table 4). Whereas for the physiotherapists 100% were in favor of these instruments, for the oncologists it was 76.9% and for the “other” group 62.9%. As regard to the specific tests that are considered the most useful according to the free text responses, the 6-minute walking test was mentioned fifteen times, NRS six times, and the timed get and go was mentioned four times.

### Program design

The preferences were split between a fully individualized modular program or a combination of partly standardized (core modules) and partly individualized according to specific needs (Table 5). These results were modified by language region of the participants. Conspicuously, in the Latin-speaking regions while over half of the respondents were in favor of a combined program, in the D-CH only one third were of this opinion. When the same question was set in the context of oncological rehabilitation after completion of the acute phase, similar results were achieved with just under half of the respondents choosing a fully individualized program and half choosing a combination program of partially standardized and partly individualized modules.

**Table 5:** Program design

How should the program be structured if rehabilitation begins during acute oncological treatment? According to language region.	German speaking	French speaking	Italian speaking	Total
	*n* = 43	*n* = 13	*n* = 15	*n* = 71
An individual modular program	55.8%	38.5%	46.7%	50.7%
A standardized program	4.7%	7.7%	0.0%	4.2%
A standardized program with core modules + further modules according to individual requirements	34.9%	53.8%	53.3%	42.3%
I do not know	4.7%	0.0%	0.0%	2.8%
*P* = 0.81				
How should the program be structured if rehabilitation begins after completion of acute oncological treatment? According to medical specialty.	Oncologist	Physiotherapist	Other	Grand Total
	*n* = 13	*n* = 23	*n* = 35	*n* = 71
An individual modular program	46.2%	26.1%	54.3%	43.7%
A standardized program	0.0%	8.7%	5.7%	5.6%
A standardized program with core modules + further modules according to individual requirements	46.2%	65.2%	37.1%	47.9%
Other	7.7%	0.0%	0.0%	1.4%
I do not know	0.0%	0.0%	2.9%	1.4%
*P* = 0.31				

The core modules that were considered by most participants to belong to a standardized program were physiotherapy (66%) and exercise and sports therapy (76%). In the cases of nutritional counseling and psychotherapy/psycho-oncology, approximately half of the respondents considered these modules as part of a standardized program. The remaining modules were considered by the majority to be elective and based on individual need. These included social counseling and support, complementary medicine, occupational therapy, sexual counseling, creative therapy (painting and music therapy), speech and swallowing therapy, and pastoral care. There were no apparent deviations in any of the answers to these questions from the respondents according to their specialty or language region.

The quantity of ambulatory rehabilitation that a patient can cope with during the oncological treatment was quantified in the questionnaire during and after the acute phases of treatment. For patients who have ongoing acute treatment, the median number of modules was two per week corresponding to a median duration of rehabilitation of 120 min per week. Following completion of the acute treatment, the median number of rehabilitation modules that can be completed increased from two to four modules per week with a total duration of OR which increased overall from 120 to 180 min. The median of 4 was consistent across all specialties and language regions apart from the oncologists where the median was 3.

The desired total length in weeks of the ambulatory physiotherapy was investigated in the survey for both an individual modular program and for a standardized program. For both an individual modular program and for a standardized program the median length was recorded at 12 weeks. This was strongly supported and consistent for all specialties and language regions.

The definition of an interdisciplinary ambulatory program and the minimal number of modules to fulfill the definition was surveyed. Overall, for an ambulatory program to be considered interdisciplinary, the median number of modules on offer should be at least four, with the patient completing at least 3 modules during their OR program.

### Financing and certification

Overall, there was no clearly preferred system among the different specialties (Table 6).

**Table 6:** Financing and certification. According to medical specialty

	Oncologist	Physiotherapist	Other	Total
	*n* = 13	*n* = 23	*n* = 35	*n* = 71
How should the billing of services look like?				
Individual billing	23.1%	21.7%	31.4%	26.8%
Flat rate billing	23.1%	30.4%	22.9%	25.4%
Combination of above	38.5%	30.4%	28.6%	31.0%
I do not know	15.4%	17.4%	17.1%	16.9%
*P* = 0.97				
What would be the advantage of certification? (multiple selection possible)				
Recognition	28.1%	34.5%	40.2%	36.0%
Quality assurance	25.0%	31.0%	36.6%	32.6%
Standardization of performance/programs	28.1%	24.1%	18.3%	22.1%
No advantage	6.3%	5.2%	1.2%	3.5%
Other	9.4%	1.7%	1.2%	2.9%
I do not know	3.1%	3.4%	2.4%	2.9%

The pattern of responses was quite similar across the three language reasons. The only apparent outlier was in the French-speaking region, where the flat rate billing model was preferred by more than half of this group.

The survey checked on the need for certification of the OR program. Almost all respondents (94.2%) were in favor of a certification of the ambulatory program for oncology; reasons being recognition by patients and stakeholders, guarantee of quality of the program, and delivery of a standardized program, but also improved program financing and reimbursement.

## Discussion

The objectives of rehabilitation are the improvement of functional limitations and improvement of activities in everyday life, in the world of work and in participation. To support these objectives, SW!SS REHA has defined quality and performance criteria to govern the set up and deployment of multidisciplinary OR programs and interventions in Switzerland [10, 11].

In contrast to cardiac rehabilitation where the patients are more homogeneous, cancer patients are heterogeneous and a standard program cannot so easily be uniformly applied [12]. Therefore, it seems reasonable to have a more flexible program and fix the threshold for mandatory modules quite low so that patients in need are not excluded, can gain access to the programs and subsequently benefit from the interventions. Physiotherapy and physical exercise have been proven to be of value and are recommended for all ambulatory OR patients in the international literature [12]. The international data supports the fact that ambulatory OR programs are usually extensively tailored to individual patients’ needs [5]. Furthermore, when information was found on mandatory interventions, it was observed that it was solely with reference to physical activity [13].

National evidence–based cancer rehabilitation guidelines in the Netherlands are considered the most advanced [14]. They can provide some important insights for desired future developments in Switzerland. Based on these guidelines, cancer rehabilitation refers solely to rehabilitation medicine, which is an outpatient interdisciplinary treatment aimed at maximizing autonomy and participation of (former) cancer patients who have multiple and interrelated problems as a result of having cancer and/or the treatment of it. Importantly, cancer rehabilitation does not comprise mono- or multidisciplinary interventions for patients who have single or unrelated functional problems, although this service is offered in primary care to cancer patients. Therefore, with regard to the actual SW!SS REHA guidelines and to ensure delivery of true multidisciplinary OR, the evidence suggests that the minimum number of interventions should not be reduced below the currently recommended four interventions. However, all cancer patients should continue to receive some form of mandatory physical activity and if they require interrelated multidisciplinary OR therapy a minimum of three additional non-physical activity interventions should be decided based upon individual medical need.

The duration of the OR is not fixed in the SW!SS REHA criteria. According to survey participants, the program should last on average 12 weeks, independent of the type of program and whether it is an individual modular or a standard program. Based on the information that could be gathered from the international literature research with data available for eight of the 15 countries [8], outpatient cancer rehabilitation program durations varied between 3 weeks in Germany [15] and up to 30 weeks in some of the Canadian programs [6], the average duration being 9.5 ± 4.5 weeks. In the Netherlands, the duration also varied between 8 and 12 weeks [14].

Among the program prerequisites in the SW!SS REHA criteria are a minimum of 10 treatment units per week per patient. Regarding the frequency of rehabilitation interventions (or sessions) per week, a high degree of variation between countries was identified. Nevertheless, the average intensity seemed to be around 2 to 4 sessions/interventions per week [16–18], lending support to the feedback from the ambulatory OR centers in Switzerland that 10 units per week is not feasible for both practical and medical reasons. In addition to the number of treatment units, we also assessed the duration or the time per week spent with OR measures. This depended on when the OR starts and was shorter for patients who still receive active treatment (120 min) compared to those who already finished the treatment (180 min).

Another area of divergence between the SW!SS REHA criteria and the survey results concerns the leadership and organization of the multidisciplinary OR program. From the guided discussion [8], it was learned that the SW!SS REHA requirement to have at least 2 years rehabilitation experience, when the program director is not a PMR specialist, is a resource challenge. The survey results confirmed that oncologists are the preferred choice to lead the outpatient program because of their better understanding of the future recovery potential of the oncology patients and their expertise to asses patients’ ability to undergo multidisciplinary OR. The international literature review indicates that in the nine countries where data was available on this criterion [8], usually the PMR specialist or other selected member within the rehabilitation team, e.g., physiotherapist and not an oncologist with rehabilitation experience led the program. Therefore, if oncologists are to continue to perform this function in Switzerland, this experience requirement may need to be softened to a minimum one year of experience, as indicated by the survey results, in order to avoid potential rationing of OR. In addition to the quantitative requirements expressed in number of years of OR experience, the precise definition of what exact experience is indicated with the term “OR experience” still needs be clarified.

The SW!SS REHA criteria stipulate that a coordinating function is mandatory but do not specify which specialty should be accountable for ensuring the patient coordination in the program. The Swiss survey results indicate a strong preference for a rehabilitation team. Moreover, the role of the coordinating function should be clarified in the SW!SS REHA criteria. The coordinator should be someone from the multi-professional rehabilitation team, with clear responsibility and triage and decision-making processes. According to the international literature review [8], there is usually a coordinator and the function varies by country.

Financing of OR programs is not addressed in the SW!SS REHA criteria. During the guided discussion, guaranteed financing was identified as a conducive factor for the successful implementation of an OR program, although there was no clear consensus on whether one financing model would be more conducive to a successful OR program than another one. For this reason, the most frequent models were tested for approval in the survey. This showed that of the three main options, including individual fee for service billing, flat rate billing, or combination of both, there was no clear difference in favor of one model. For those advocating a combination of flat fee for core modules with a fee for service approach for additional models, flat rate billing was foreseen for the physiotherapy and exercise/sports therapy interventions, inclusive of coverage of the cost of coordinating all additional elective modules, as coordination time and costs for the OR program also need to be financed.

Certification is currently not possible for the centers offering OR programs, because they cannot currently completely fulfill all the SW!SS REHA criteria. According to the survey, 75% of respondents consider certification as advantageous. The international literature research did not reveal any certification as such in the fifteen countries investigated. What could be observed was that some countries seemed to have quality standards for their programs. In Luxembourg [18, 19], after the start of the program every two and a half years, there should be an external audit and, in the Netherlands, this should be once every 5 years [14]. The potential benefit of certification or quality standards and its implications for Switzerland requires further analysis and investigation.

The Swiss national study on interdisciplinary ambulatory OR provides a thorough basis to design an evidence-based ambulatory OR program building upon and where necessary adapting the SW!SS REHA guidelines with the relevant actors. The online survey confirms that many of the SW!SS REHA quality and performance criteria fit with the actual or desired OR programs in Switzerland. Other criteria might need to be added, adapted or removed.

The guided discussion had indicated that there may be important differences in the approach to OR among the different language regions or specialists [8]. The results of the survey were therefore stratified by three specialty groups and the three language regions. With just one exception concerning the value of specialty specific instruments, none of the other tests performed reached statistical significance and therefore do not provide hard evidence of an interaction or dependence. Therefore, it is considered valid to develop single national quality and performance criteria for all regions in Switzerland.

The overall response rate was 49% and was lower in some groups such as the centers in F-CH or in the mixed group of specialists “other.” It would be valuable to follow-up with a sample of non-responders, to investigate the underlying reasons behind their non-response. In the context of the above non-responder results, this could give valuable insights as to what extent non-responding members of the “other” group, e.g., nurses, occupational therapists, and social councilors, feel concerned resp. do not feel concerned by the subject of multidisciplinary ambulatory oncological rehabilitation programs and how they could be better integrated into OR programs.

The international literature review has indicated that the level of evidence supporting multidisciplinary ambulatory OR is modest [8]. There is therefore a need to generate more real-world evidence, patient-reported outcomes and health outcomes data to provide stronger evidence for the clinical and social benefits of ambulatory OR. More evidence would help ensure that more appropriate cancer patients gain access to evidence-based OR care in Switzerland. Having said this, we acknowledge that the patients’ perspective has not been considered in our study, we solely focused on the providers’ perspective. Considering the patients’ perspective though is crucial and needs to be assessed in future studies.

For clarity of focus, our survey focused on the two main phases of rehabilitation, namely ongoing acute oncological treatment phase or after completion of the acute phase. In practice, there are several phases of OR covering pretreatment rehabilitation, rehabilitation during cancer therapies, between cancer therapies, after cancer therapies and both a curative and non-curative approach. Additionally, acute oncological treatments could be further differentiated, including chemotherapy, immunotherapy, targeted oral therapies, radiotherapy, and surgeries of varying intensity with either extensive neoadjuvant radio-chemotherapy or just simple adjuvant hormone therapies. The fuller set of indications or settings should ideally be investigated with further research.

*ESAS*, Edmonton Symptom Assessment Scale; *WHODAS*, WHO Disability Assessment Schedule; *ECOG*, Eastern Cooperative Oncology Group Performance Status; *HADS*, the Hospital Anxiety and Depression Scale; *NRS*, Numerical Rating Scale

## Supplementary Information

ESM 1 (PDF 491 kb)

## Data Availability

We have full control of all primary data and agree to allow the journal to review these data if requested.
