# Supplementary material for: National study for multidisciplinary outpatient oncological rehabilitation: online survey to support revised quality and performance criteria
Source: Support Care Cancer. 2020 Dec 8;29(7):3839–47. doi: 10.1007/s00520-020-05913-z (PMC8163662; doi:10.1007/s00520-020-05913-z)
Supplement: Supplementary file 1 — (PDF 491 kb) [file 520_2020_5913_MOESM1_ESM.pdf]

Einleitung zum Fragebogen

Sehr geehrte Befragungsteilnehmerin
Sehr geehrter Befragungsteilnehmer

Ihre Meinung zur zukünftigen Auslegung der SW!SSREHA-Kriterien sowie zur Finanzierung der ambulanten onkologischen Rehabilitationsprogramme (Onko-Reha) für ambulante onkologische Patienten und Patientinnen ist für uns und die onkologische Gemeinschaft in der Schweiz sehr wichtig.

Im vergangenen Jahr haben einige von Ihnen am ersten Teil der Studie zur ambulanten onkologischen Rehabilitation teilgenommen und die Umfrage zur aktuellen Situation in der Schweiz beantwortet. Dadurch haben wir jetzt einen klaren Überblick darüber, was zum jetzigen Zeitpunkt angeboten und umgesetzt wird. Vielen Dank dafür.

Ziel dieses zweiten Teils der Studie ist es, auf der Basis der ersten Ergebnisse die Grundlage für die Ausarbeitung von modifizierten Qualitäts- und Leistungskriterien sowie eine geregelte Finanzierung für die Schweiz zu schaffen. Dazu benötigen wir erneut Ihren Input als Expertin und Experte in Onkologie und onkologischer Rehabilitation, um Ihre Bedürfnisse bei der Gestaltung eines optimalen onkologischen Rehabilitationsprogramms für ambulante Patienten und Patientinnen zu erfassen.

Da wir beabsichtigen, eine quantitative Analyse aller an der ambulanten onkologischen Rehabilitation beteiligten Fachgebiete durchzuführen, ist es wichtig, dass wir möglichst viele vollständige Antworten erhalten. Wenn Sie sich bei der Beantwortung einer der Fragen für Ihr Fachgebiet nicht angesprochen fühlen oder keine Antwort wissen, kreuzen Sie bitte "Ich weiss es nicht" an.

Das Ausfüllen der Online-Befragung wird 20 bis 30 Minuten in Anspruch nehmen.

Sobald die Befragung ausgewertet ist, werden wir uns wieder mit Ihnen in Verbindung setzen, um Sie über die Ergebnisse und die nächsten Schritte im Prozess zu informieren.

Um die Umfrage zu starten, klicken Sie bitte auf den folgenden LINK.

Wir bitten Sie, die Umfrage bis spätestens Freitag, den 12. April 2019 online auszufüllen.

Vielen Dank im Voraus für Ihre Unterstützung!

Für das EBPI der Universität Zürich

Prof. Dr. oec. troph. Sabine Rohrmann, MPH
Institut für Epidemiologie, Biostatistik und Prävention

Für die KLS:

Beate Schneider
Fachspezialistin Rehabilitation
D-Schweiz

Nicolas Sperisen
Fachspezialist Rehabilitation
Romandie & Tessin

<u>Fragebogen zur ambulanten onkologischen Rehabilitation</u>

Fachgebiet der Teilnehmer/Teilnehmerin (zur Auswahl, abrufbar)

Ergotherapie
Ernährungsberatung
Komplementärmedizin
Leitung/Koordination/Administration
Onkologie
Pflege
Physiotherapie
Psychotherapie/Psychologie/Psychoonkologie
Schmerztherapie
Seelsorge
Sozialberatung
Sport-, Bewegungstherapie
Stomatotherapie
Andere

Zentrum der Teilnehmer/Teilnehmerin (zur Auswahl, abrufbar)

<u>Leitung und Organisation</u>

1. Wer sollte die medizinische Leitung der ambulanten onkologischen Rehabilitation haben? (Mehrfach-Auswahl möglich)

- ☐ Onkologin/Onkologe
- ☐ AIM (Arzt/Ärztin für allgemeine innere Medizin)
- ☐ PMR (Arzt/Ärztin für physikalische und medizinische Rehabilitation)
- ☐ Andere Fachärzte/Fachärztinnen (freier Text)

  ____
- ☐ Ich weiss es nicht

2a. Wieviel Rehabilitations-Erfahrung ist notwendig, um die medizinische Leitung zu übernehmen, wenn nicht PMR? (Auswahl)

- ☐ Keine notwendig
- ☐ Bis 1 Jahr
- ☐ Zwischen 1 und 2 Jahre
- ☐ 2 Jahre oder mehr
- ☐ Ich weiss es nicht

2b. Bitte begründen Sie kurz Ihre Antwort (freier Text)

3a. Wer ist aus Ihrer Sicht am besten geeignet, die ambulante Onko-Reha zu koordinieren? (Auswahl)

- ☐ Arzt/Ärztin Onkologie, AIM, PMR oder andere medizinische Fachrichtung
- ☐ Physiotherapeut/Physiotherapeutin
- ☐ (Onkologie-)Pflege
- ☐ Gemeinsam im multiprofessionellen Reha-Team
- ☐ Andere (freier Text)

____

- ☐ Ich weiss es nicht

3b. Bitte begründen Sie kurz Ihre Antwort (freier Text)

4. Wie sieht der optimale Informationsfluss zwischen den Fachpersonen aus? (Mehrfach-Auswahl möglich)

- ☐ KLS Reha-Logbuch (Alle Informationen beim Patienten)
- ☐ Dokumente
    - o Via E-Mail
    - o Formalisierter Bericht
    - o elektronisches Patienten-Dossier
    - o Andere (freier Text) ______________________
- ☐ Andere (freier Text) ___________________________
- ☐ Ich weiss es nicht

5. Wie oft sollte eine Reha-Teambesprechung stattfinden? (Auswahl)

- ☐ Einmal pro Woche
- ☐ Einmal pro Monat
- ☐ Einmal pro Programm
- ☐ Reha-Teambesprechung nicht notwendig
- ☐ Andere (freier Text) ___________________________

Zielgruppe und Screening Instrument

6. Patienten/Patientinnen mit welchen Indikationen (Defiziten/Problemen) brauchen nach Ihrer Einschätzung eine Rehabilitation? Bitte geben Sie aus Ihrer Sicht die **4 wichtigsten Indikationen an,** für die Aufnahme in eine interdisziplinäre ambulante onkologische Rehabilitation **(Voraussetzung).**

- ☐ Funktionalität/Mobilität im täglichen Leben eingeschränkt
- ☐ Körperliche Leistungsfähigkeit/Aktivität im Alltag eingeschränkt
- ☐ Besondere Ernährungssituation vorhanden
- ☐ Emotionale Probleme und eingeschränkte Lebensqualität
- ☐ soziale, berufliche oder finanzielle Situation eingeschränkt
- ☐ Handlungsfähigkeit im persönlichen, häuslichen und/oder beruflichen Umfeld eingeschränkt
- ☐ Lebensqualität als Folge der Krankheit oder Therapien eingeschränkt
- ☐ Störung des emotionalen/spirituellen Gleichgewichts
- ☐ Eine besondere Pflegesituation vorhanden
- ☐ Umgang mit Krankheits- oder Therapiefolgen erschwert

- ☐ Ausgeprägte Schmerzen vorhanden
- ☐ Müdigkeit beeinträchtigt den Alltag
- ☐ Fragen/Anliegen zur Sexualität vorhanden
- ☐ Sprach- oder Schluckprobleme vorhanden
- ☐ Andere (freier Text)_________________
- ☐ Ich weiss es nicht

7. Wer sollte die Gesamt-Rehabilitationsziele mit dem Patienten/der Patientin festlegen? (Auswahl)

- ☐ Arzt/Ärztin Onkologie, AIM, PMR oder andere medizinische Fachrichtung
- ☐ Physiotherapeut/ Physiotherapeutin
- ☐ Onkologie-Pflegefachperson
- ☐ Gemeinsam im multiprofessionellen Reha-Team

- ☐ Andere (freier Text) ______________________________
- ☐ Ich weiss es nicht

8. Welche generischen Instrumente zur Erhebung des Reha-Bedarfs halten Sie für sinnvoll? (Mehrfach-Auswahl möglich)

- ☐ ESAS-Score, allenfalls WHODAS II
- ☐ ECOG/Karnofsky oder adaptierter ECOG
- ☐ Evaluation der Funktionellen Leistungsfähigkeit (EFL)
- ☐ Distress-Thermometer
- ☐ Weitere Assessments
- ☐ Falls weitere, welche (freier Text) ___________________________
- ☐ Ich weiss es nicht

9. Halten Sie fachspezifische Instrumente zur Messung von Verlauf und Zielerreichung in Ihrem Fachgebiet für sinnvoll z. B. 6-Minuten Gehtest, Timed get up and go, HADS, NRS, etc.

- ☐ Ja
- ☐ Nein
- ☐ Ich weiss es nicht

9b. Wenn ja, welche fachspezifischen Instrumente? (freier Text)

Das Programm

10a. Wie sollte das Programm aufgebaut sein, wenn die Rehabilitation **während der akuten onkologischen** Behandlung beginnt? (Auswahl)

- ☐ [1]Ein individuelles modulares Programm (jeder Teilnehmer nach einem gemeinsam ermittelten Rehabilitationsbedarf)
- ☐ [2]Ein standardisiertes Programm (alle Teilnehmer machen dasselbe Programm)
- ☐ Ein standardisiertes Programm mit Kernmodulen + weiteren Modulen nach individuellem Bedarf
- ☐ Andere (freier Text) ______________________
- ☐ Ich weiss es nicht

10b. Wie sollte das Programm aufgebaut sein, wenn die Rehabilitation **nach Abschluss der akuten onkologischen** Behandlung beginnt? (Auswahl)

- ☐ Ein individuelles modulares Programm (jeder Teilnehmer nach einem gemeinsam ermittelten Rehabilitationsbedarf)
- ☐ Ein standardisiertes Programm (alle Teilnehmer machen dasselbe Programm)
- ☐ Ein standardisiertes Programm mit Kernmodulen + weiteren Modulen nach individuellem Bedarf
- ☐ Andere (freier Text) ______________________
- ☐ Ich weiss es nicht

[1] Individuelle modulare Programme: Programme sind in der Regel länger, werden individuell festgelegt und die Reha-Massnahmen (Module) folgen meistens nacheinander

[2] Standardisierte Programme: Programme sind kürzer, alle Patienten und Patientinnen haben die gleichen Kernmodule (+ zusätzliche Massnahmen bei Bedarf) und die Reha-Massnahmen (Module) finden meistens gleichzeitig statt.

11. Welche (Kern-)Module sollten in einem standardisierten ambulanten onkologischen Rehabilitationsprogramm immer enthalten sein bzw. welche Module sollten ergänzend dazu nach individuellem Bedarf des Patienten/der Patientinnen „wählbar“ sein (nur eine Antwort pro Zeile)

	Standardisiertes Programm (Kernmodule)	Module nach individuellem Bedarf	Ich weiss es nicht
Physiotherapie			
Bewegungs- & Sporttherapie			
Ernährungsberatung			
Psychotherapie & Psychoonkologie			
Soziale Beratung & Unterstützung			
Komplementärmedizin			
Ergotherapie			
Sexualberatung			
Gestalterische Therapie, Maltherapie, Musiktherapie			
Logopädie, Schlucktherapie			
Seelsorge			
Pflege			
Andere (freier Text): ____			

12. Wie viele Module (verschiedene Fachbereiche) pro Woche sind **insgesamt** für Patienten/Patientinnen **machbar**?

- während der akuten onkologischen Behandlung _ Module/Woche
- nach Abschluss der akuten onkologischen Behandlung _ Module/Woche

13. Wie viele Minuten pro Woche sind **insgesamt** für Patienten/Patientinnen **machbar**?

- während der akuten onkologischen Behandlung _ Minuten/Woche
- nach Abschluss der akuten onkologischen Behandlung _ Minuten/Woche

14. Wie lange sollte das gesamte ambulante Reha-Programm aus Ihrer Sicht durchschnittlich in Wochen dauern, um wirksam zu sein?

- Für ein individuelles modulares Programm _Wochen
- Für ein standardisiertes Programm _Wochen

15. Wie viele Module (verschiedene Fachbereiche) müssen mindestens angeboten werden für ein ambulantes interdisziplinäres onkologisches Rehabilitationsprogramm?
- mindestens _ (Anzahl)

16. Wie viele Module (verschiedene Fachbereiche) muss ein Patient/eine Patientin mindestens belegen für ein ambulantes interdisziplinäres onkologisches Rehabilitationsprogramm?
- mindestens _ (Anzahl)

Prozesse

17. Wer sollten die Zuweiser sein? (Mehrfach-Auswahl möglich)

- ☐ Hausarzt/Hausärztin
- ☐ Gynäkologe/Gynäkologin
- ☐ Arzt/Ärztin Onkologie, AIM, PMR
- ☐ Arzt/Ärztin anderer medizinische Fachrichtung
- ☐ Patient/Patientin selbst
- ☐ Kantonale Krebsliga (KKL)
- ☐ Andere (freier Text) ___________________________
- ☐ Ich weiss es nicht

18. Wer sollte über die Aufnahme und den Eintritt in ein ambulantes onkologisches Rehabilitations-programm entscheiden? (Auswahl)

- ☐ Arzt/Ärztin Onkologie, AIM, PMR oder andere medizinische Fachrichtung
- ☐ Onko-Reha-Team
- ☐ Andere nicht ärztliche Fachdisziplin
- ☐ Wenn andere nicht ärztliche Fachdisziplin, dann welche? (freier Text)____
- ☐ Ich weiss es nicht

19. Was sind aus Ihrer Sicht förderliche Faktoren für die Umsetzung eines ambulanten onkologischen Rehabilitationsprogramms? (freier Text)

Finanzierung

20a. Wie sollte die Abrechnung der Leistungen aussehen? (Auswahl)

- ☐ Einzelabrechnungen
- ☐ Pauschale Abrechnung
    - Wenn ja, welche Module sollten enthalten sein (freier Text)
- ☐ Kombination der beiden oben
- ☐ Andere (freier Text)___________________________
- ☐ Ich weiss es nicht

20b. Bitte begründen Sie kurz Ihre Antwort (freier Text)

Zertifizierung

21. Was wäre der Vorteil einer Zertifizierung? (Mehrfach-Auswahl möglich)

- ☐ Anerkennung (Patienten/Patientinnen, Behörden, Kostenträger, Fachpersonen usw.)
- ☐ Qualitätssicherung
- ☐ Standardisierung der Leistungen und der Programme
- ☐ Kein Vorteil
- ☐ Andere (freier Text)_____________________________
- ☐ Ich weiss nicht

22. Haben Sie weitere Bemerkungen oder Kommentare zu dem Thema oder dem Fragebogen?

VIELEN HERZLICHEN DANK FÜR IHRE TEILNAHME AN DER BEFRAGUNG!
